# Fear, Isolation, and Invisibility during the COVID-19 Pandemic: A Qualitative Study of Adults with Physical Disabilities in Marginalized Communities in Southeastern Michigan in the United States

**DOI:** 10.3390/disabilities2010010

**Published:** 2021

**Authors:** Lisa Reber, Jodi M. Kreschmer, Gina L. DeShong, Michelle A. Meade

**Affiliations:** 1 Department of Physical Medicine and Rehabilitation, University of Michigan, Ann Arbor, MI 48108, USA; 2 The Disability Network, Flint, MI 48507, USA

**Keywords:** fear, isolation, invisibility, caregivers, marginalized communities, COVID-19, physical disability, qualitative, interviews, rehabilitation

## Abstract

This study examines the initial impact of the COVID-19 pandemic on adults with physical disabilities from marginalized communities in southeastern Michigan, one of the early pandemic epicenters in the United States. A purposeful sample of fifteen adults with moderate to severe physical disabilities were recruited, taking part in individual remote semi-structured qualitative interviews, which were recorded, transcribed, and coded for emergent themes using a thematic approach to coding and analysis. Three interrelated, overarching themes emerged: fear, feelings of isolation, and a sense of being invisible. These were identified in the contexts of health and healthcare, home care assistance, and access to resources. The findings help illuminate the experiences of those from socioeconomically and racially marginalized communities, populations that are often “always already” vulnerable. Participant narratives made visible the negative impact of the pandemic on physical and mental health as well as the lack of accommodations available. They showed that participants were faced with a dilemma between engaging in risky behavior to have their needs met or avoiding risk and not have those needs met. This knowledge can expand awareness and appreciation of how social, economic, and political systems impact adults with physical disabilities in lower-income and racially diverse communities and provide guidance in designing future clinical and emergency response policies.

## 1. Introduction

In early March 2020, the World Health Organization (WHO) declared the spread of coronavirus disease 2019 (COVID-19) a pandemic. Wayne County in southeast Michigan soon followed New York City as an epicenter in the United States (US). By early April, Michigan had almost 19,000 confirmed cases and 845 deaths [1]. As of 14 January 2021, there were 14,325 deaths in Michigan, ranking it eighth in the country.

The pandemic may appear to be indiscriminate; however, some groups are at far greater risk for severe complications and negative impact than others. The distribution of these risks within society are socially and structurally determined and include age, sex, socioeconomic status, racial identities, and disability [2–4]. Poverty and race/ethnicity are also key epidemiological factors that put people at risk for a severe case of COVID-19 [5,6]. In the United States, race accounts for 24% of COVID-related deaths [3]. These compounding vulnerabilities often result in entire communities being disproportionally impacted when crisis hits. Recent survey research on the impact of the pandemic on individuals with disabilities documented concerns about access to personal care assistance, a lack of transportation, and social isolation [7–11]. These issues have also been prevalent in the news, commentaries, and editorials [12–15]. Existing survey research notes participation by only a limited number of people of color [7–11]. Even more limited is the intersection between disability and race/ethnicity, which has been addressed in the news and public discussion but has received limited attention by researchers [16–19]. Moreover, regardless of race/ethnicity or socioeconomic background, there has been limited use of qualitative methods to examine the impact on COVID on people with physical disabilities.

Individuals with physical disabilities can be dynamic actors who, despite living with the risk factors associated with their disabilities, have found ways to navigate their environment and maintain important life activities. In providing one of the first glimpses of the lived experiences of adults with physical disabilities from socioeconomically and racially/ethnically marginalized communities during the early days of the COVID-19 pandemic, this study targeted those whom we believed would have both awareness of the challenges that exist as well as insight about the programs, information, and resources that were more helpful for achieving positive outcomes. Drawing from remotely conducted interviews with those living or working in lower-income and primarily African American communities in Metro Flint and Detroit in southeastern Michigan, this study aims to identify the barriers and facilitators that impacted their experiences of the pandemic.

## 2. Design and Methods

This study employed a qualitative research design to explore the subjective perceptions and experiences of active, engaged adults with long-term physical disabilities in lower-income Black/African American majority communities. This approach was determined to be the most effective for understanding how participants interpreted the impact of COVID-19 on their lives and to obtain in-depth, thick descriptions of their experiences [20,21]. The study was approved by the University of Michigan’s institutional review board (#HUM00152705).

### 2.1. Recruitment and Screening

A purposive sampling approach was used to identify participants and data collection was limited to two months, April and May 2020, to capture early experiences of the pandemic. Research shows that when a purposive sampling approach of a relatively homogeneous population is taken coding becomes fairly stable after only twelve interviews and in some cases fewer [22,23]. Inclusion criteria required participants be 18 years with a moderate to severe physical disability of at least five years in zip codes identified as lower income in Metro Flint or Detroit in southeastern Michigan. In the US, a two-person household is considered low-income if it is less than 80% of the area median income (AMI). In the city of Detroit, the threshold is USD 50,240; very low-income is USD 31,400 or below [24]. Flint and Detroit are majority African American, 80% and 60%, respectively. Thus, an additional factor impacting recruitment was obtaining a sample that reflected these racial demographics. In addition, participants were selected, in part, based on their responses to the WHODAS 2.0 measure for disability and health as well as supplemental questions about activity level and participation outside of the home. The aim was to prioritize engagement of participants with higher levels of functional impairment and greater level of engagement with personal, family, and community activities and participation.

Potential participants were identified with the help of disability and community organizations and community organizers to whom information about the study was emailed. These groups then shared this information within their communities. Two Community Liaisons—both African American and one with a physical disability—also assisted. Individuals who were interested in participating called and were screened by a research assistant.

### 2.2. Data Collection

The interview protocol was semi-structured, designed to be a rough guide with open-ended questions about the pandemic. A broad range of additional questions related to social and community supports, healthcare access, and the built and attitudinal environments were included to elicit COVID-related experiences and insights participants might have overlooked. Participants directed conversations and topics not addressed were prompted. Interviews were conducted one-on-one remotely by phone or video by the lead author (L.R.) and recorded with a Zoom Communication tool. Consent for recording and interviewing was obtained from participants at the start of each interview. Two hours were allocated for each interview to allow more salient information to emerge [23]. Participants received a USD 100 debit card for participating. As much effort as possible was made to remove information that might make participants traceable from the data presented [25], and participants were given pseudonyms (L.R.). The lead author (L.R.) de-identified all data collected and documents were saved on password-secure computers and networks.

### 2.3. Study Sample

The final sample (*N* = 15) included nine Black/African Americans (60%), four Whites (27%), one Middle Eastern American (6%), and one Native American (6%) interviewed in the pandemic’s initial months (April and May 2020). Ages ranged from early 20s through mid-70s, five were female and ten male, and all participants had moderate to severe physical disabilities (see Table 1). Eight participants were either activists or worked in disability organizations. As a result, their interviews included examples from their own lives as well as those with whom they engaged. Only half of the participants were willing to provide their annual household income. Five were USD 29,000 or below and two were USD 39,000 or below. Based on participants’ descriptions of their homes and communities during interviews, we estimate that three more participants were USD 29,000 or below and the remaining were above USD 39,000. Interviews averaged an hour and forty minutes.

### 2.4. Analysis

All interviews were transcribed verbatim by a professional service using an encrypted database and transfers. The first author (L.R.) confirmed accuracy through careful review of transcripts with recordings. Transcripts were then de-identified and sections related to COVID extracted from the narratives. Data analysis was guided by a constructivist perspective, one which views experiences, meanings, and realities as socially produced [26,27]. Using NVivo data analysis software, the first author approached coding inductively, using a line-by-line approach to identify what people were doing and what was happening (L.R.) [28]. Analysis also drew on the work of Braun and Clarke’s reflexive thematic analysis approach, which supports attending to people’s views and experiences as well as the examination of data that are richly detailed and nuanced [26]. The process of analysis was iterative and continually evolved. It moved between the identification of codes and patterns in the data and the development of themes and sub-themes and sorting of associated narratives (L.R. and senior author M.A.M.) [26]. Themes, categories, and interpretations were rigorously discussed, developed, and revised until consensus was achieved (L.R., J.M.K., G.L.D., and M.A.M.).

To maintain the trustworthiness and validity of interpretation, several steps were taken. A strength of our study and central to establishing the groundwork for obtaining trustworthiness were weekly meetings between the first author’s (L.R.), a white woman without a physical disability, and the Community Liaisons. These meetings helped to ensure awareness of issues as related to recruitment, the implementation of focus groups and interviews, and the interpretation of narratives and their regional context. In addition, a researcher with a physical disability (J.M.K.) and one of the African American Liaisons (G.L.D.) were active members of the research team. Descriptive validity was checked to ensure participant excerpts were correctly used and accurately reported (L.R., J.M.K., and G.L.D.) [29,30]. Trustworthiness in the interpretation of data was provided through the rich complementary perspectives of our diverse research team, which made space for discussions and critiques of our positionalities and how they influenced meaning-making [28,31].

## 3. Results

Three overarching themes were generated: feelings and emotional reactions, the contexts in which the feelings and emotions were identified, and how needs were accommodated. Within the overarching theme of feelings and emotional reactions, four sub-themes were identified: fear, feelings of isolation, a sense of being invisible, and experiencing little emotional distress and disruption to life. The three subthemes were identified related to the second overarching theme, the contexts in which participants’ feeling and emotions were experienced. These were health and healthcare, home care assistance, and access to resources. The third overarching theme, accommodations of needs, has no sub-themes.

### 3.1. Feelings and Emotional Reactions

#### 3.1.1. Fear and Perceptions of Risk

Fear was the most notable emotion expressed by participants. They displayed a wide range of risk perceptions and tolerance, revealing their diverging perceptions of and feelings about the pandemic. Some, such as Sam, said that they were not concerned about the pandemic: “It really doesn’t [impact my life]”. In some cases, as Laura explained, adults with physical disabilities had to weigh their needs against potential risks or fears they might have had:

They’re just saying, ‘I value what this person can give me, more than I value the consequences.’ [… and] ‘Hey, I’m aware that there’s lots of other ways the virus can get, you know, contracted, but I need this person to help me, you know, take a shower today. I need this person to go get my groceries.’

Some participants were extremely concerned and expressed their fear overtly. Grady said, “I just—I’m really—I’m really afraid, because people are saying that people keep—the numbers keep going up!” Erica, a participant and community disability organizer, said that people with disabilities “talk about being shut-in, and not like being able to get out of the house—and being fearful of getting out of the house”. The pandemic, she added, “is much worse [for us.] We rely on so many things in order for us to live our lives, you know, abundantly [ … ] It’s scary. [ … ] Your fear, it can push you into depression”. Participants, such as Michael, worried about shopping and medical needs and having to take public transportation: “It’s very difficult now, very difficult now, because I–I’m afraid to get on the bus with all of this COVID going around”.

For some, fear came from the ambiguity created by the pandemic. Meridian explained, “but mostly, it’s–it can impact you just mentally, a lot. Just the–the fear of change, and how it’s going to play out, how long it’s going to be”. Similarly, Carnise stated:

How they’re going to integrate people back into work, and so, my concern is would they make sure that all the infection rate is completely down? Because you got the president talking about he wants to reopen, like, yesterday, so that causes some anxiety, and especially in people like me that’s more at risk.

#### 3.1.2. Sense of Isolation

Feelings of isolation were also a commonly shared experience during the pandemic. Nine participants addressed it. Some said adults with disabilities experience isolation more intensely than those without disabilities. Laura described the cumulative effect of isolation and how this felt like an intensification of something that is an enduring component of their lives: “[The pandemic is] further isolating people. So, for people that felt isolated before, this is … almost like a deeper dive into isolation”. Narratives also revealed the critical role that medical practitioners and rehabilitation activities played in the socialization of adults with disabilities. Ramon said, “I used to go therapy, like, four days a week, but of course, I can’t do that … I don’t really–really talk to nobody”. In addition, Tavell’s statement reveals the anxiety and fear that isolation can produce: “It’s like being in jail. Now, here, I’m–I’m–I’m–and I’m here only because of the pandemic, … So, I–I–I’m not able to get out, I don’t like being closed in”.

#### 3.1.3. Feeling Invisible

Feelings of invisibility were the third notable feeling. When speaking about their experiences during the pandemic (or the experiences of those with whom they worked), six participants identified a sense of being forgotten. Erica’s comment reflects a belief that the needs of adults with disabilities had been forgotten and the fear this engendered: “If everybody else is shutting down, oh my God, if they’re shutting down, where does that leave me?!” This included, as Hazel explained, feeling they had been overlooked by the government: “The Governor has decreed that certain things are not priority issues. Well, yeah, on one hand they’re not priority for some people [ … ] the whole reaction to this virus has left out people with chronic illnesses”. In addition, as Grady added, “they’ve just not really addressed, us people. […] I knew that was going to happen, because I live in Michigan, and they don’t really care much about handicapped people”. Laura explained that the people with disabilities with whom she worked felt overlooked by society as a whole: “I think part of [why the pandemic is worse] is just because we still feel unseen, right? We still feel like we’re not a part of the collective human experience”.

#### 3.1.4. Experiencing Little Emotional Distress and Disruption to Life

Although most participants spoke about the pandemic and some at quite length, a small number of participants said very little about it because they thought the pandemic had made little disruption to their lives. Asked if their lives had changed because of the pandemic, Sam said, “Quite honestly, it doesn’t”. In addition, Kaleem said, “Hmm, no. Actually, everything is about the same”. In these cases, participants spoke about their daily routine having been minimally impacted. These individuals reported varying contexts and needs; one participant, Ramon, already had COVID and had a full-time caregiver while another did not require caregivers; both had limited excursions outside of their home prior to the onset of the pandemic. These participants also tended to express less concern about contracting the virus. Sam had no concern: “I’m not all that concerned”. Kaleem spoke about trying to keep his immune system up and Ramon mentioned the interruption to his physical therapy. However, neither seemed especially concerned.

### 3.2. Contexts Where Fear, Isolation, and Invisiblity Were Experienced

#### 3.2.1. Health and Healthcare

Fear, isolation, and a sense of invisibility were associated with the barriers participants encountered. One such barrier, which several participants referenced, was in the context of health and healthcare and in relationship to rehabilitation and medical services. Tavell said, “I don’t receive massage therapy anymore [or] go to strength training any longer. —Core training [provided] relief [and without it, I feel] soreness and pain coming back”. In addition, as Meridian said, “I’m not getting out of the house as much, I find myself being a little more stiff, or I might have pain”. Steve expressed concern about being neglected in the hospital should his personal care assistant be unable to accompany him: “When a person–if they’re paralyzed, and they’re–they’re–they’re recovering, there’s not time to have people sitting in your room, you know, waiting on you–A person like me, I need 24-hour care. It’s about survival, really”. Hazel stated that the regular check-ups she required were often canceled because they were not considered “essential”, and contended that postponing necessary medical care would have far-reaching repercussions that the government had not considered: “I think we’re going to have this boomerang effect of having neglected our disability population. I think when this is over, I’ll be able to regain what I’ve lost, but I think there’s probably people who won’t”.

#### 3.2.2. Personal Homecare Assistance

Of the 15 participants, 4 lived alone, 7 had access to personal care assistance who were family members or roommates, and 3 had 24-hour paid care. One participant, who required multiple assistants, expressed concern with them entering her home and devised a plan to avoid this: “If it became a pandemic, then this is the plan of action. I’ll leave [my] house [ … and move in with one of] my caregiver[s] and his aunt”. In contrast, another participant was frustrated that her house cleaning service was now refusing to come. Hazel said, “I can’t really do–clean the house myself. You know, because I’m in a chair I can’t get–there’s lots of places that I just can’t get to”. She argued that she should have more to fear than those who would come to her home because she never left it. Tavell, too, did not understand: “They can close the door while they’re vacuuming. How can I contract it? I–I just don’t understand that”.

#### 3.2.3. Access to Resources

Access to resources, such as food, personal care supplies, and personal protective equipment (PPE), was a general concern. In this context, several participants talked about barriers to accessing groceries. Erica said, “If you have to stay in the house because you’re susceptible to what’s going on out in the public, then what do you do?!” Hazel spoke about a local business with whom she was a long-term customer forgetting about her needs.

These places have been providing these services and known I have a disability all this time, and there’s been no planning for the people they’ve been serving all along to somehow say, okay, these are our customers that we’ve had, we have to prioritize them.

Other participants were unable to access delivery because they lacked the money to pay for it. If they could have, they lacked the technology or technical knowledge required to request delivery online. Eric and Laura also encountered this among those with whom they worked. Laura said, “They might not be able to afford–[or] even know how to navigate the internet [for ordering groceries]”.

### 3.3. How Needs Were Accommodated

The subthemes presented in the overarching theme on contexts shown above reflect participants’ inability to access accommodations. This third overarching theme identifies when participant needs were met. Only one clearly articulated his ability to gain them. Through contacts with state officials, one participant obtained approval to move in with just one of his state-funded personal care assistants, allowing him to reduce the risk created by multiple bodies entering his home: “I went through [a governmental office] that I know. We talked. [ … ] I put them in touch with [the non-profit], which is my fiduciary so that they could explain [my situation] to them better”. This participant also benefited from Michigan’s Executive Order No. 2020–48 that suspended the rule requiring physical presence during a public body meeting [32]: “I was on a [name] board, and because of bed rest, I wasn’t able to do my obligation of being there to vote physically. [ … ] People now can conduct state board meetings from their home, in Michigan, at least”.

Some needs were met by organizers, such as Erica, as part of the non-profit organization she founded for adults with disabilities. After hearing of the need, she and others gained funding and organized a delivery service for those in their organization who were unable to afford delivery. Erica’s organization also organized remote group sessions: “We would have everybody on this call, and we would just talk to each other, make sure everybody is doing good. [ … ] Just the camaraderie with one another”. She added that they would also have “experts” join their sessions to impart information. This information, however, was not always sound. Erica reported one expert telling the group that drinking hot water would help “flush the virus [ … ] down into your gut”.

## 4. Discussion

The aim of this study was to center the experiences that adults with long-term physical disabilities from lower-income and racially marginalized communities encountered in the early months of the COVID-19 pandemic, despite living active, engaged lives prior to the pandemic. Focusing on the intersecting experiences of this population is important because of the challenges created by the unequal distribution of risk often created by more limited economic opportunities and racial inequalities [33], inequalities which the pandemic compounded [34]. When learning about how to protect oneself from the virus, socio-economic inequalities can potentially impact the quality and type of experts to which groups have access. This may have been the case for the participant in this study who described her others being told by an “expert” that they could “flush the virus [ … ] down into your gut”. There were reliable sources of information available to participants, such as the weekly webinars produced by the Healthy Flint Research Coordinating Center and information provided by the United States’ Centre for Disease Control (CDC); however, whether participates accessed such resources was unknown to the research team. As Frederick and Shifrer [35] (p. 201) argue, the experiences of individuals with disability are “co-constituted with the system of racial stratification”, resulting in it being “experienced disproportionately” by racially marginalized groups.

In our study, participant narratives revealed fear and a sense of isolation and being invisible. These reactions were most often associated with barriers to health and healthcare, homecare assistance, and access to resources. Fear, for most participants, was an overarching facet of their lives. Their fears were driven by the challenges they were experiencing because of the pandemic, a belief that they were being overlooked by society, and that their needs would go unmet. The need to isolate alone in their homes reinforced their sense of invisibility, deepening the isolation and sense of invisibility many experienced prior to the pandemic. Participant narratives made visible the need to balance risk and need, the lack of accommodations, and the negative impact on physical and mental health. They showed that most of the participants felt they would have to engage in risky behavior to have their needs met or avoid risk and not have those needs met. Risk and need determined the impact of their experiences and demonstrates the importance of accommodations as a moderating factor between the two.

However, while most participants spoke about the concerns and challenges created by the pandemic, a few exceptions were noted and identified in the fourth subtheme, experiencing little emotional distress and disruption to life. The participants referenced in this section said that the pandemic had done little to alter their lives. Interestingly, in comparison, the descriptions of daily life provided by most participants also had not differed significantly. The difference between the two groups—those who indicated a change to their routine and those who did not—was in what they said (or did not say) about the pandemic; those who indicated little change did not articulate as much fear and concern or talk as extensively about the challenges they were facing.

Fear has also been identified in other COVID-19 related research. In interviews conducted with older adults, fear was found to be related to uncertainty about the virus and becoming infected [36] and in focus groups composed of individuals with different disabilities fear was discussed in relation to medical rationing [37]. Two survey studies of individuals with disabilities did not address fear specifically; however, they did find a high prevalence of anxiety and depression [7] and perceived stress [11], two reactions that share many properties with fear [38,39]. A fear that did not emerge in our study was fear of losing structure to one’s daily life [36]. Interestingly, in contrast, a survey study conducted with adults with physical disabilities in Italy found little psychological distress resulting from the pandemic [40]. The authors reasoned that the isolation created by the pandemic stay-at-home orders had mild impact because of the population’s prior and ongoing experience with isolation. It may also be due to many other factors, such as differences in public health messaging, the timing of the spread, and socio-economic differences in the populations. A key difference between our sample population and the studies discussed above are their racial demographics. While, when stated, the sample population in the above studies ranged between 63% and 88% white, our sample (*N* = 15) reflected a more racially marginalized population: 60% black, 13% other, and 27% white. In addition, the socioeconomic demographics of our sample population likely differed, too.

Interestingly, survey research where participant experiences were most similar to ours was among adults with low vision or blindness [8]. Although its participants were predominantly white, expressions of fear were equally prevalent. Adults with low vision or blindness also endorsed concerns about social isolation as well as feelings of depression and loneliness due to social distancing [8]. Where our findings differed were in the nuance our data revealed; narratives suggests that feelings of loneliness and isolation may also result from a sense of invisibility and not having needs acknowledged. In addition, the fear that can emerge from having to engage in risky behavior to meet needs during the pandemic did not emerge in the other studies. Finally, participants in our study also discussed barriers to accessing resources. African Americans, in particular, spoke about being unable to access food delivery services, technology, or safe transportation due to financial limitations.

### 4.1. Healthcare Challenges

When findings from our study are interpreted through the lens of the Model of Healthcare Disparities and Disability (MDHH), a framework that extends the *International Classification of Functioning, Disability, and Health* [41], attention is brought to the structural issues of ableism, racism, and socioeconomic disparities. The framework focuses the lens on the unconscious biases that can inform healthcare providers’ beliefs, which can lead to discrimination and a reduction in healthcare quality and access [42]. Other research on the impact of COVID-19 has found that the majority of adults with disabilities are concerned with healthcare-related issues, including access to care, healthcare rationing and disruptions in care [7–9,37]. These themes were also addressed by our participants who explained in detail the repercussions of lost treatment and therapies and the fear of having no accommodations made for them should they require hospitalization. For African Americans with disabilities, who were already at risk of healthcare rationing, barriers to access and fears are compounded [43,44].

In the state of Michigan, awareness of concerns with rationing and discrimination in the delivery of care and medical resources came in early April 2020 with Executive Order No. 2020–64 [45]. This Order was designed to ensure against such biases. Yet, people with disabilities continue to have legitimate concerns about rationed healthcare and access [4,14,46–50]. While the Executive Order made provisions for sign language interpreters, it lacked other essential provisions, such as ensuring access to one’s personal care assistants if hospitalized. As participant narratives illustrated, accommodations tend to come to those who have the power and resources to pressure change [51] or simply to those who are lucky. In the absence of policy, accommodations are seen as special treatment rather than a right, and for those in marginalized groups, the discrimination encountered is often even greater [52].

### 4.2. Limitations

Our small select sample limits our ability to draw comparisons with other groups, racially and socio-economically. While their narratives provide evidence of how the pandemic was experienced by them, they did not explicitly say—nor were they asked—how or why their experiences differed from white or affluent adults with physical disabilities. In addition, although we were able to draw conclusions about participants financial conditions based on their narratives, indicating that the majority were lower income, we lacked household income data for all participants to support this. However, rather than being problematic, our findings are likely an underestimation of the severity of the issue given potentially higher incomes among some in the sample.

## 5. Conclusions

The results of our qualitative study expand existing knowledge and offer new insights into how the pandemic impacted physical and psychological health of adults with physical disabilities from marginalized communities. Our participants’ experiences are familiar. They support prior scholarship demonstrating that challenges such as invisibility, healthcare, and resources are problematic for adults with disabilities [53]. However, our narratives also suggest that these challenges are different, in that the pandemic appears to have intensified prior challenges. As our participant, Laura, stated about those with whom she worked, “For people that felt isolated before, this is … almost like a deeper dive into isolation”. This illustration also reflects how the universality of certain experiences, such as isolation, can differ from those without disabilities.

As this study begins to demonstrate through participant narratives, qualitative research can illuminate how the pandemic differentially impacts adults with physical disabilities. It can also provide “a deeper dive” into understanding how race/ethnicity and socioeconomic status can further marginalize a population that is “always already” vulnerable. This knowledge can expand awareness of how social, economic, and political systems are structured and integrated into future clinical guidelines and emergency response policies. Specifically, it can inform policy makers of the challenges faced when accommodations are not made for at risk populations, when adults with disabilities from diverse communities are not included in the decision-making process, and when key resources and services to adults with disabilities are deemed non-essential. By making the invisible visible, the needs of certain members of society, their care, and unique health concerns, and the resources they require can be more adequately addressed.

Policy solutions have been and continue to be developed. These recommendations address the rights and dignity of persons with disabilities and the structural disparities that produced them during the COVID-19 pandemic [37,54,55]. The importance of being aware of and prepared to manage the needs of individuals with disabilities during natural disasters have been identified [56,57]. Hospitals and health systems are beginning to develop policies that adapt policies to be more inclusive of the needs of individuals with disabilities, including providing exceptions to visitor policies and allowing essential caregivers to be present; moreover, they are working to strengthen interdisciplinary care by including rehabilitation psychologists, who are well-positioned to advocate for the needs of people with disabilities, especially those who are further marginalized because of race and socioeconomic status [49,58]. Recommendations have also been outlined to address challenges that occur for individuals with disabilities during stay-at-home orders and in response to reduced access to caregivers and other needed healthcare resources [37,59,60]. Solutions are there. Policymakers at all levels—organizational, state, and national—need only act.

## Data Availability Statement:

Following curation, the data presented in this study will be available here: https://www.icpsr.umich.edu/web/ICPSR/series/1740.

## Figures and Tables

**Table 1.** Participant characteristics.

Alias	Diagnosis/Disability	Race/Ethnicity	Gender	Age
Carnise	SCI: Paraplegia	Black/African American	Male	30s
Meridian	Rheumatoid Arthritis	Black/African American	Female	40s
Robert	Neuropathy	Black/African American	Male	50s
Michael	Spina Bifida	Black/African American	Male	70s
Ramon	Lower Limb Amputation (double)	Black/African American	Male	40s
Tavell	SCI: Paraplegia	Black/African American	Male	60s
Grady	Cerebral Palsy	Black/African American	Male	20s
Kaleem	Amputation of toes	Black/African American	Male	40s
Erica	SCI: Paraplegia	Black/African American	Female	60s
Aisha	Cerebral Palsy	Middle Eastern/White	Female	20s
Hakan	Spina Bifida	Native American/White	Male	40s
Steve	SCI: Tetraplegia	White	Male	40s
Sam	Lower Limb Amputation (single)	White	Male	60s
Hazel	Hip dysplasia	White	Female	60s
Laura	Cerebral Palsy	White	Female	30s
